# Intrinsic emergence and modulation of sex‐specific dominance reversals in threshold traits

**DOI:** 10.1111/evo.14563

**Published:** 2022-07-20

**Authors:** Jane M. Reid

**Affiliations:** ^1^ Centre for Biodiversity Dynamics NTNU Trondheim Norway; ^2^ School of Biological Sciences University of Aberdeen Aberdeen UK

**Keywords:** Liability, sex‐specific dominance reversal, sexual antagonism, sexual conflict, sexual dimorphism, threshold trait

## Abstract

Sex‐specific dominance reversals (SSDRs) in fitness‐related traits, where heterozygotes' phenotypes resemble those of alternative homozygotes in females versus males, can simultaneously maintain genetic variation in fitness and resolve sexual conflict and thereby shape key evolutionary outcomes. However, the full implications of SSDRs will depend on how they arise and the resulting potential for evolutionary, ecological and environmental modulation. Recent field and laboratory studies have demonstrated SSDRs in threshold(‐like) traits with dichotomous or competitive phenotypic outcomes, implying that such traits could promote the emergence of SSDRs. However, such possibilities have not been explicitly examined. I show how phenotypic SSDRs can readily emerge in threshold traits given genetic architectures involving large‐effect loci alongside sexual dimorphism in the mean and variance in polygenic liability. I also show how multilocus SSDRs can arise in line‐cross experiments, especially given competitive reproductive systems that generate nonlinear fitness outcomes. SSDRs can consequently emerge in threshold(‐like) traits as functions of sexual antagonism, sexual dimorphism and reproductive systems, even with purely additive underlying genetic effects. Accordingly, I identify theoretical and empirical advances that are now required to discern the basis and occurrence of SSDRs in nature, probe forms of (co‐)evolutionary, ecological and environmental modulation, and evaluate net impacts on sexual conflict.

Core ambitions in evolutionary biology are to identify key processes that maintain genetic variation in fitness and that shape the outcome of evolutionary sexual conflict (Johnson and Barton 2005; Bonduriansky and Chenoweth 2009; Connallon and Clark 2010, 2014; Arnqvist 2011; Connallon 2015; Hendry et al. 2018; Plesnar‐Bielak and Łukasiewicz 2021). Observations that magnitudes of standing genetic variation in fitness (and major fitness components) can substantially exceed those expected solely due to mutation‐selection balance imply that some forms of balancing selection must act to maintain polymorphisms (Johnson and Barton 2005; Charlesworth 2015; Connallon and Chenoweth 2019). While sexual conflict resulting from sexually antagonistic selection can in principle be resolved through evolution of sexual dimorphism, such outcomes depend on genetic architectures of focal traits, including sex‐specific additive and nonadditive genetic effects and (co)variances (Lande 1980; Connallon and Clark 2010; Arnqvist et al. 2014; Wyman and Rowe 2014). Accordingly, overarching objectives are to identify interacting processes and architectures that can jointly generate balancing selection and facilitate the emergence of sexual dimorphism and to understand how such processes and architectures can themselves arise or be constrained (Bonduriansky and Chenoweth 2009; Connallon and Clark 2010, 2014; Connallon 2015; Llaurens et al. 2017; Grieshop and Arnqvist 2018; Ruzicka et al. 2019; Kaufmann et al. 2021; van der Bijl and Mank 2021).

In this context, sex‐specific dominance reversals (SSDRs) are of direct interest because they constitute one key mechanism that could both maintain genetic variation and ameliorate sexual conflict (Fry 2010; Arnqvist 2011; Arnqvist et al. 2014; Grieshop and Arnqvist 2018; Connallon and Chenoweth 2019; Ruzicka et al. 2019; Grieshop et al. 2021). SSDRs are defined as occurring when heterozygotes' phenotypes resemble the phenotypes of alternative homozygotes in females versus males (Fig. 1). Such SSDRs allow expression of differing sex‐specific optimal phenotypes given the same heterozygous genotype at a focal locus (Fig. 1). This can in turn generate net heterozygote advantage at the population level, which can contribute to maintaining genetic variation (i.e., through net balancing selection, Fig. 1). Such SSDRs could therefore both defuse sexual antagonism and maintain potential for future evolution (Fry 2010; Arnqvist 2011; reviewed by Connallon and Chenoweth 2019; Grieshop et al. 2021). However, in general, such impacts will depend on the frequency of occurrence and magnitude of effect of SSDRs and hence on the circumstances under which SSDRs can actually arise, evolve and be modulated in nature (as with dominance relationships more generally, Billiard et al. 2021).

**Figure 1:**
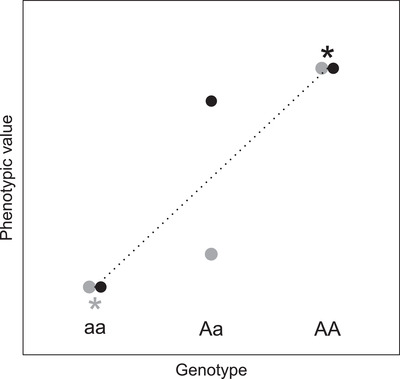
Basic illustration of (partial) sex‐specific dominance reversal (SSDR), generating (partial) phenotypic optimization in both sexes. Given two possible alleles (a and A) at a focal large‐effect locus, the aa homozygote has a lower phenotypic value than the AA homozygote in both sexes (sexes shown in black and gray). The phenotypic value of the Aa heterozygote is closer to that of the AA homozygote in the black sex and to that of the aa homozygote in the gray sex, constituting SSDR. If high and low phenotypic values lead to higher fitness in the black and gray sexes respectively (asterisks, implying sexually antagonistic selection), then there will be net heterozygote advantage across the population, generating balancing selection that can help maintain genetic variation. The dashed line highlights the expected phenotypic value of the Aa heterozygote given purely additive allelic effects. The illustrated scenario shows symmetrical SSDR with no phenotypic sexual dimorphism in either homozygote. However, more generally, the two sexes could show different degrees of partial or complete dominance with some degree of sexual dimorphism in the homozygotes. Sex‐specific dominance, but not SSDR, would arise if phenotypic values for the Aa heterozygotes are above (or below) the additive expectation in both sexes, but to different degrees. SSDR is typically defined on the phenotypic scale (as depicted). By analogy, genome‐wide rather than single‐locus SSDRs could arise if heterozygous offspring of crosses between (relatively) homozygous parental lines show mean phenotypes that resemble different parental lines in the two sexes.

Fundamental questions of whether dominance of beneficial versus detrimental alleles can directly evolve and/or simply arises as an intrinsic property of nonlinear genotype‐phenotype (or genotype‐fitness) maps have been widely considered and historically generated considerable controversy. One key contention was that, since dominance manifests in heterozygotes, a relatively high frequency of heterozygosity is required to generate appreciable selection on dominance and hence any possible dominance evolution, yet sufficient heterozygosity may not generally arise (arguments summarized by Otto and Bourguet 1999; Manna et al. 2011; Spencer and Priest 2016; Connallon and Chenoweth 2019; Billiard et al. 2021). However, recent population genetic theory shows that dominance can in principle evolve in circumstances where some additional process generates or maintains heterozygosity (Otto and Bourguet 1999; Billiard et al. 2021). This includes evolution of SSDRs given sexually antagonistic selection (Spencer and Priest 2016). Here, sexual antagonism can initially maintain sufficient genetic variation (and hence heterozygosity) at focal large‐effect loci to allow invasion of sex‐specific dominance modifiers, which effectively reduce sexual conflict and further maintain genetic variation (Spencer and Priest 2016).

Meanwhile, it has also been highlighted that intrinsic SSDRs can emerge if genotype‐fitness maps for both sexes are nonlinear and, specifically, concave around each sex's optimum. Given strong sexually antagonistic selection at a focal locus such that opposite homozygotes have higher fitness in females versus males, sex‐specific fitness values for heterozygotes can then be geometrically closer to each sex‐specific maximum even given purely additive underlying allelic effects (Fry 2010; reviewed and illustrated by Connallon and Chenoweth 2019). This scenario concurs with the general points that any nonlinear genotype‐phenotype map can generate intrinsic dominance (Gilchrist and Nijhout 2001; Vasseur et al. 2019) and that recessivity in detrimental small‐effect mutations can arise given smooth nonlinear fitness landscapes (e.g., given stabilizing selection across underlying traits, Manna et al. 2011). Hence, overall, the points that evolved and/or intrinsic SSDRs could in principle exist are now well substantiated (Grieshop and Arnqvist 2018; Connallon and Chenoweth 2019; Grieshop et al. 2021).

Indeed, four major empirical studies have now demonstrated SSDRs in key fitness‐related traits in disparate systems. These four studies concern maturation in Atlantic salmon (*Salmo salar*, Barson et al. 2015); occurrence of anadromy (i.e., sea migration) in rainbow trout (*Oncorhynchus mykiss*, Pearse et al. 2019); survival through bacterial exposure in *Drosophila melanogaster* (Geeta Arun et al. 2021); and competitive reproductive success in seed beetles (*Callosobruchus maculatus*, Grieshop and Arnqvist 2018). They provide striking evidence of SSDRs involving heterozygosity at known large‐effect loci or genomic inversions (Barson et al. 2015; Pearse et al. 2019) or given polygenic heterozygosity generated through heroic efforts with experimental evolution and/or line crosses (Grieshop and Arnqvist 2018; Geeta Arun et al. 2021).

However, while these four studies demonstrate manifestations of SSDRs, they do not focus on investigating how such SSDRs could or do arise. This is reasonable; simply demonstrating SSDRs in fitness‐related traits in wild or wild‐derived systems represents a notable advance, while probing their basis requires further challenging investigations (Pearse et al. 2019; Geeta Arun et al. 2021; Grieshop et al. 2021). However, some insights into underlying mechanisms, specifically the degrees to which observed phenotypic SSDRs represent explicit genetic dominance reversals versus intrinsic properties of nonlinear genotype‐phenotype or genotype‐fitness maps, will ultimately be required to fully understand key forces that maintain genetic variation in fitness and resolve sexual conflict in nature. This is especially true when SSDRs are revealed by experimental evolution and/or line crosses; such approaches may be highly effective in demonstrating potential for SSDRs but may not necessarily imply that observed effect sizes routinely arise or hence substantively shape evolutionary outcomes in the wild.

Here, jointly considering the four empirical studies (Barson et al. 2015; Grieshop and Arnqvist 2018; Pearse et al. 2019; Geeta Arun et al. 2021) can help develop conceptual frameworks and hypotheses. In particular, all four studies concern threshold or threshold‐like traits, defined here as focal phenotypes that are manifested as dichotomous or competitive outcomes (further explained below). This is notable because threshold(‐like) traits (as opposed to traits that are directly expressed and continuously distributed on observed phenotypic scales) are not typically the predominant focus of work in quantitative genetics or experimental evolution or of explicit theory on (sex‐specific) dominance evolution. The observation that all four empirical studies that demonstrate SSDRs concern threshold(‐like) traits consequently raises interesting questions of whether such traits have properties that foster evolution and/or expression of SSDRs involving large‐effect loci and/or polygenic variation and hence how such traits could play key roles in maintaining genetic variation and resolving sexual conflict in nature.

To address these questions, I first summarize pertinent properties of threshold(‐like) traits. I then demonstrate how these properties can readily generate phenotypic SSDRs that arise as intrinsic consequences of sexual dimorphism and/or competition without necessarily requiring either direct SSDRs in underlying allelic effects or directly concave genotype‐fitness maps. I achieve these objectives using illustrative caricatures of traits, quantitative genetic architectures and study designs reported in the four recent empirical studies (Barson et al. 2015; Grieshop and Arnqvist 2018; Pearse et al. 2019; Geeta Arun et al. 2021). I thereby use these studies as inspiration to consider how SSDRs could arise but do not imply that outlined scenarios necessarily apply in their focal systems. Finally, I highlight how explicitly considering the properties of threshold(‐like) traits opens new theoretical and empirical routes to examining the dynamics of SSDRs and their impacts on evolutionary outcomes in nature.

## Fundamental Properties of Threshold Traits

**Figure 2:**
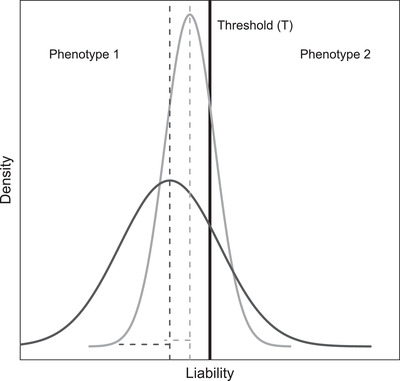
Basic concept of a threshold trait. Individuals have liabilities (x‐axis) comprising genetic and environmental effects that translate into expression of phenotype 2 versus phenotype 1 when above versus below the threshold (T, black vertical line), generating an intrinsically nonlinear genotype‐phenotype map. Dark and light gray curves show hypothetical distributions of liabilities for two populations or groups (which could be sexes) with the same number of individuals in each group (i.e., same area under each curve). The mean (vertical dashed lines) and standard deviation (horizontal dashed lines) of the liability distributions both differ between the two groups (the dark gray group has a lower mean and greater standard deviation and hence greater variance). Nevertheless, the proportion of individuals who expresses phenotype 2 (i.e., the relative area under each curve that exceeds the threshold), and hence the mean phenotype, is the same for both groups (0.21 in the depicted example). Hence, sexual dimorphism in mean liability will not necessarily translate into sexual dimorphism in observed phenotype. Mathematical treatments are in Supporting Information S1

The threshold trait concept has long been established in quantitative genetics as a route to rationalizing and predicting the dynamics of dichotomous phenotypes underpinned by highly polygenic genetic architectures. In brief, individuals are envisaged to have latent ‘liabilities’, which can comprise additive and/or nonadditive genetic and environmental effects and are assumed to be continuously distributed across individuals. Individual liability values translate into expression of alternative discrete phenotypes when above or below some threshold (Figure 2, Falconer and Mackay 1996, Ch. 18; Roff 1996; Lynch and Walsh 1998, Ch. 25; Reid and Acker 2022). The threshold trait concept therefore explicitly invokes a highly nonlinear genotype‐phenotype map and hence a nonlinear genotype‐fitness map if resulting dichotomous phenotypes substantively impact fitness.

While the basic threshold trait construction envisages a steep fixed threshold that generates entirely discrete phenotypes (Figure 2, Falconer and Mackay 1996; Lynch and Walsh 1998), the concept can be broadened to encompass shallower threshold slopes that could yield partial trait expression and which could themselves evolve (Chevin and Lande 2013). Broadly threshold‐like properties can consequently arise for fitness components that are not intrinsically phenotypically dichotomous but that emerge from competitive interactions with some degree of “winner takes all.” For example, competition for reproductive resources, matings or fertilizations can result in substantial variance in outcomes, even with relatively little variance in underlying trait values, if ‘winning’ individuals monopolise disproportionate shares (as observed in numerous systems, e.g., Dodson et al. 2013; Laturney et al. 2018; Parker 2020; see Discussion). The values of competing individuals must then be exceeded to achieve substantial reproductive success.

Since nonlinear genotype‐phenotype maps generally generate intrinsic dominance (Billiard et al. 2021), it is immediately plausible that threshold(‐like) traits could induce such effects. Indeed, it is well established that threshold traits transform effects that are strictly additive on underlying liability scales into nonadditive effects on observed phenotypic scales (Gianola 1982; Lynch and Walsh 1998; de Villemereuil 2018). There can therefore be substantial “cryptic” genetic variation in liability, which has little or no immediate effect on phenotype, on either side of the threshold (Roff 1996, 1998; Reid and Acker 2022). However, despite these properties, intrinsic dominance and SSDRs more specifically have predominantly been formally theoretically considered in the context of smooth fitness surfaces, with little explicit consideration of threshold(‐like) traits (Fry 2010; Manna et al. 2011; Connallon and Chenoweth 2019; Vasseur et al. 2019; but see Gilchrist and Nijhout 2001 for treatments of diffusion‐gradient‐threshold models). This omission is perhaps surprising since Wright (1934) originally postulated the threshold trait concept as a parsimonious explanation for otherwise puzzling and inconsistent patterns of apparent inheritance and dominance (as observed for guinea pig digit numbers).

Furthermore, the threshold trait construction also fundamentally implies that the mean observed phenotype for any group of individuals depends not only on mean liability (relative to the threshold) but also on the variance in liability. This is because the mean and variance jointly define the proportion of individuals whose liabilities exceed the threshold and hence express the alternative phenotype (Figure 2, Supporting Information S1, Falconer and Mackay 1996). Hence, in the context of sex‐specific effects, the observed degree of phenotypic sexual dimorphism in a threshold trait jointly depends on the degrees of sexual dimorphism in the mean and the variance in liability. Accordingly, sexual dimorphism in mean liability might or might not translate into sexual dimorphism in phenotype, depending on the degree of sexual dimorphism in the variance and on the distances of the sex‐specific mean liabilities from the threshold (Figure 2, Supporting Information S1).

Given these well‐established properties, the potential for threshold(‐like) traits to generate SSDRs on observed phenotypic scales, with or without explicit genetic SSDRs acting on underlying liability scales, can be outlined with broad reference to the four recent empirical studies.

## Linking from Empirical Studies to Concepts of SSDRs in Threshold(‐Like) Traits

### SSDRs INVOLVING LARGE‐EFFECT LOCI: SCENARIOS BASED ON SALMONIDS

Theory on SSDRs, involving either evolution of direct dominance modifiers or intrinsic effects of nonlinear fitness landscapes, primarily envisages large‐effect loci that detectably affect fitness (Fry 2010; Spencer and Priest 2016). Correspondingly, Barson et al. (2015) and Pearse et al. (2019) demonstrate SSDRs involving large‐effect loci that affect related life‐history traits in salmonids: maturation in Atlantic salmon and anadromy in rainbow trout. Both traits are commonly sexually dimorphic; males mature earlier and are less anadromous than females on average. Such dimorphisms likely result from sexually antagonistic selection arising because reproductive success is more strongly positively related to body size in females than males. This drives female‐specific selection for prolonged growth, anadromy and later maturation, which trades off against an increased probability of prereproductive mortality (Barson et al. 2015; Czorlich et al. 2018; Pearse et al. 2019). Meanwhile, the relatively undifferentiated sex chromosomes of salmonids have been suggested to inhibit sequestration of sexually antagonistic genes through sex linkage, generating interest in identifying additional mechanisms that could resolve sexual conflict (Pearse et al. 2019).

In salmon, genome‐wide association studies using relatively high‐density SNP data revealed a large‐effect locus, VGLL3, where alternative alleles substantially affect the occurrence of maturation and hence result in maturation age in both sexes (Barson et al. 2015). Genome construction and SNP‐based interrogation in trout then revealed a double‐inversion supergene that affects the occurrence of anadromy (Pearse et al. 2019). In both cases, field data yielded evidence of SSDRs where heterozygotes show mean maturation ages or probabilities of anadromy that are to some degree closer to the alternative homozygotes in females versus males (e.g., Fig. 1; Barson et al. 2015; Pearse et al. 2019). However, maturation and anadromy in salmonids are also highly polygenic heritable traits affected by numerous loci with medium or small effects (e.g., Hecht et al. 2013; Weinstein et al. 2019; Sinclair‐Waters et al. 2020). Hence, they can be appropriately conceptualized as sexually dimorphic threshold traits (Dodson et al. 2013; Debes et al. 2021), where substantial standing polygenic variation in liability can exist alongside polymorphic large‐effect loci.

**Figure 3:**
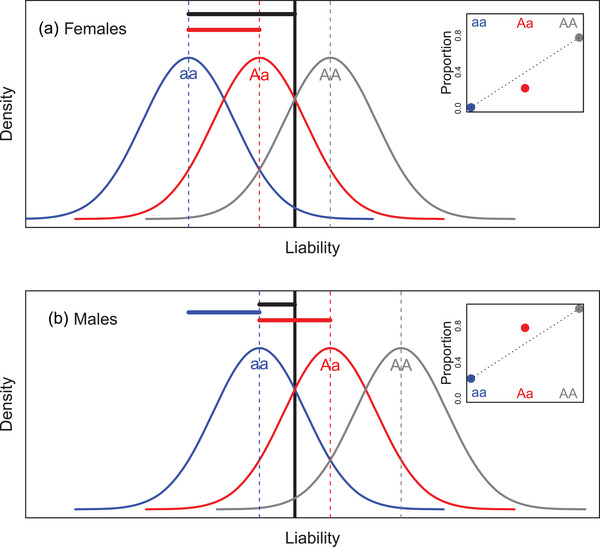
Illustration of emergence of (partial) sex‐specific dominance reversal (SSDR) in a threshold trait with sexual dimorphism in mean baseline liability and a large‐effect locus with purely additive allelic effects on liability. Blue and gray curves show the population‐wide distributions of liabilities for the two alternative homozygotes at the large‐effect locus (aa and AA, assuming an additional polygenic architecture) in (A) females and (B) males. Red curves show the population‐wide distributions of liabilities for the heterozygotes (Aa), assuming additive effects of the alternative alleles (i.e., codominance) on the liability scale that are the same in both sexes. The black vertical line denotes the threshold, above which the alternative phenotype is expressed. Accordingly, inset panels show the proportions of individuals of each homozygote (blue and gray) and the heterozygote (red) that express the alternative phenotype. Dotted lines link the proportions for the two homozygotes, visualizing that the proportions for the heterozygotes lie below versus above the additive expectations in females versus males, constituting SSDR (e.g., Fig. 1). In the main figures, vertical dashed lines denote mean liabilities. Black horizontal lines denote the distances from each sex‐specific mean for the lower homozygote (i.e., blue vertical line) to the threshold. The blue horizontal line highlights the degree of sexual dimorphism in mean liability (i.e., distance between the blue vertical lines for males versus females). Red horizontal lines highlight the additive effect of the alternative allele at the large‐effect locus (here, the same in both sexes). Illustrated liability distributions are Gaussian, but this is not essential to generate SSDRs (Supporting Information S3). Parameter values for the illustrated example are shown in Supporting Information S2

Simple illustrations then show how such genetic architectures could readily generate phenotypic SSDRs. To see this, first consider that females have relatively low mean baseline liabilities to mature at a particular timepoint, with a population‐wide distribution that scarcely spans the threshold (e.g., blue curve on Fig. 3A). Meanwhile, males have higher mean baseline liabilities with identical variance, meaning that the population‐wide distribution substantially spans the threshold (e.g., blue curve on Fig. 3B). Consequently, some males will mature now, but most females will not, meaning that sexual dimorphism in mean liability translates into partial phenotypic sexual dimorphism (Fig. 3).

Then, we can consider an alternative allele at a large‐effect locus that increases mean liability equally in both sexes. Population‐wide distributions of liabilities of homozygotes for the alternative allele (i.e., genotype AA instead of baseline aa) could then substantially exceed the threshold in both sexes, meaning that most males and females will mature now, potentially still with some phenotypic sexual dimorphism (e.g., gray curves on Fig. 3).

Regarding SSDRs, the key question then concerns the locations of the sex‐specific distributions of liabilities of heterozygotes (i.e., genotype Aa) at the large‐effect locus relative to the threshold and the resulting sex‐specific frequencies of the alternative phenotypes. Here, Figure 3 illustrates how SSDRs can readily emerge, even given purely additive effects (i.e., codominance) of the large‐effect alleles on the liability scale. This scenario occurs when the increase in mean liability due to one copy of the alternative allele is sufficient to cause most of the population‐wide liability distribution to exceed the threshold in males but not females (e.g., red curves on Fig. 3B versus 3A). Mean heterozygote phenotype (i.e., the proportion of Aa individuals who express the alternative phenotype) is then closer to that for the baseline (late maturing or anadromous) homozygote in females and the alternative (early maturing) homozygote in males, representing phenotypic SSDR (e.g., inset panels on Fig. 3).

Given this scenario, Figure 3 illustrates how phenotypic SSDRs emerge from the combination of three properties: the deviations of the two sex‐specific mean baseline liabilities from the threshold (shown by the two black horizontal lines), which together define the degree of sexual dimorphism in baseline liability (blue horizontal line) and its translation into sexual dimorphism in phenotype; and the additive effect size of the alternative allele at the large‐effect locus (red horizontal lines). Diverse forms of symmetrical or asymmetrical partial or complete SSDR can consequently emerge, depending on the degree to which the three properties cause the mean liabilities for the heterozygotes and alternative homozygotes to lie on opposite sides of the threshold in the two sexes (mathematical derivations in Supporting Information S1).

**Figure 4:**
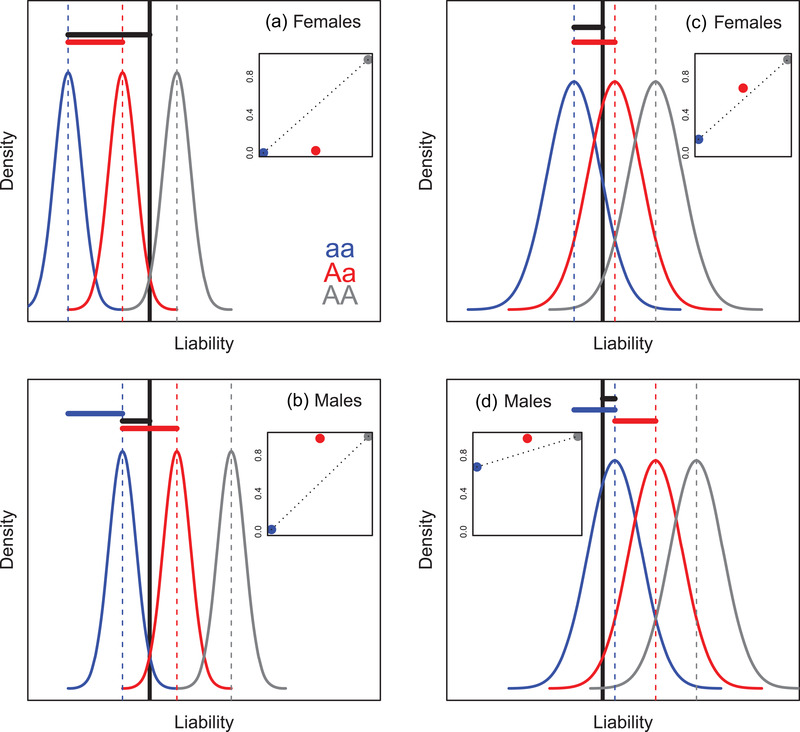
Illustrations of emergence of (A,B) complete phenotypic sex‐specific dominance reversal (SSDR) or (C,D) an absence of SSDR, given a threshold trait with sexual dimorphism in mean baseline liability and a large‐effect locus with purely additive allelic effects on liability. Specifications are as for Figure 3, where blue and gray denote the alternative homozygotes (aa and AA) and red denotes the heterozygote (Aa). (A,B) Same scenario as Figure 3 except with smaller variances in liability. Liability distributions for the heterozygotes therefore fall almost entirely on opposite sides of the threshold in (A) females versus (B) males, resulting in almost complete SSDR (inset panels). (C,D) Same scenario as Figure 3 except with higher mean baseline liabilities in both (C) females and (D) males. Liability distributions for the heterozygotes therefore fall predominantly on the same side of the threshold in both sexes, resulting in no SSDR (inset panels: red points lie above the dotted lines in both sexes). Parameter values are in Supporting Information S2. Plotted y‐axis scales differ between panels A and B versus C and D.

Such SSDRs can then be further modulated by the variance in liability and by the degree of sexual dimorphism in the variance (Supporting Information S1). For example, the scenario illustrated in Figure 3 can easily generate almost complete rather than partial SSDR given smaller variance in liability in both sexes (Figure 4A and B versus Figure 3). This is because the liability distributions for the heterozygotes (red curves) then lie almost completely on opposite sides of the threshold in females versus males (Figure 4A,B). Sexual dimorphism in the variance in liability could then generate partial phenotypic dominance in one sex and complete dominance in the other.

These scenarios imply that ongoing evolution of the degree of sexual dimorphism in the mean and/or variance in liability, or simply environmental effects on the mean and/or variance and resulting phenotypic sexual dimorphism, could alter the emerging degree of phenotypic SSDR. For example, if there were less sexual dimorphism in mean baseline liability than illustrated in Figure 3 or the same degree of dimorphism but shifted relative to the threshold, then SSDR can readily disappear (e.g., Fig. 4C,D). Hence, the forms of sexual dimorphism in the mean and variance in baseline liability, and in resulting phenotypes, can effectively act as dominance modifiers on the large‐effect locus.

Indeed, temporal and/or spatial variation in sexual dimorphism in liability could readily arise in nature if the form of (sex‐specific) selection varies among environments, potentially driving evolution of (sex‐specific) plasticity and resulting phenotypic outcomes. For example, mean salmonid maturation ages and degrees of anadromy and sexual dimorphism commonly vary among populations and even among cohorts (e.g., Dodson et al. 2013; Barson et al. 2015; Pearse et al. 2019; Weinstein et al. 2019). Observed degrees of SSDR could consequently vary among populations or cohorts, even with substantial gene flow and hence likely very similar genetic architectures. Indeed, the degree of phenotypic SSDR associated with the VGLL3 genotype differed markedly between two Atlantic salmon populations despite low genetic divergence (low F_ST_, Czorlich et al. 2018). Meanwhile, the genomic inversion that showed SSDR in anadromy in a Californian trout population (Pearse et al. 2019) had no detected effect in an Alaskan population (Weinstein et al. 2019). SSDRs can consequently be environment‐ and population‐specific rather than a fixed intrinsic property of any particular large‐effect locus or biological system, and the intrinsic properties of threshold traits can readily foster such modulations. Phenotypic dominance modification can then be straightforward; it can simply result from additional genetic and/or environmental effects on liability for any threshold trait.

**Figure 5:**
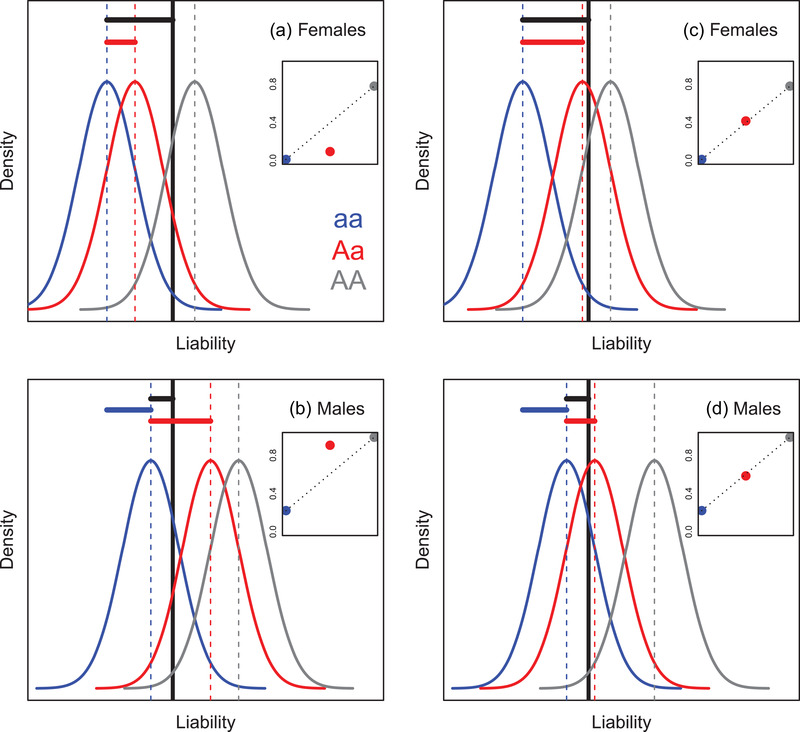
Illustrations of (A,B) strong phenotypic sex‐specific dominance reversal (SSDR) or (C,D) an absence of phenotypic SSDR, given a threshold trait with sexual dimorphism in mean liability and liability‐scale SSDR in allelic effects at a large‐effect locus. Specifications are as for Figure 3, where blue and gray denote the alternative homozygotes (aa and AA) and red denotes the heterozygote (Aa). (A, B) Same scenario as Figure 3 except that the alternative allele at the large‐effect locus shows partial liability‐scale SSDR rather than sex‐independent additivity (baseline allele is dominant in females, alternative allele is dominant in males). Strong phenotypic SSDR emerges (inset panels). (C, D) Same scenario as A, B except that the alternative allele at the large‐effect locus shows reversed partial liability‐scale SSDR (baseline allele is dominant in males, alternative allele is dominant in females). The inset panels show that the proportions of heterozygotes that exceed the threshold match the additive expectations (i.e., lie on the dotted lines in both females and males). Consequently, there is no phenotypic SSDR and, in fact, no phenotypic dominance, despite liability‐scale SSDR. On all panels, red horizontal lines highlight the sex‐specific effects of one copy of the alternative allele at the large‐effect locus relative to the baseline homozygote, encompassing allelic effect size and dominance coefficient. Parameter values are in Supporting Information S2

However, while threshold traits can readily generate phenotypic SSDRs given purely additive allelic effects on liability (Figures 3 and 4), there could in principle be direct SSDRs at the large‐effect locus that act on the liability scale (i.e., *liabilities* of heterozygotes could be closer to opposite homozygotes in the two sexes, Fig. 5, Supporting Information S1). Such liability‐scale SSDRs could translate into strong phenotypic SSDRs (Fig. 5A and B) but will not necessarily do so. Indeed, they could in principle even appear as purely additive phenotypic effects (Fig. 5C and D). This could occur if sex‐specific dominance coefficients shift the liability distributions for the heterozygotes so that the proportions of values exceeding the threshold in each sex equal the means across the two homozygotes (Fig. 5C and D). Consequently, the degree of liability‐scale SSDR cannot necessarily be directly inferred by quantifying the degree of phenotypic SSDR, or vice versa.

Nevertheless, an alternative conceptual model for the evolution of sex‐specific dominance modifiers on large‐effect loci can be postulated. Current population genetic models envisage that a focal large‐effect locus already exists and consider whether mutant (sex‐specific) dominance modifiers can invade (given some process that maintains appreciable heterozygosity at the focal locus, e.g., Otto and Bourguet 1999; Spencer and Priest 2016). However, the threshold trait scenario implies that this logic could potentially be reversed: we could assume that a potential dominance modifier (e.g., sexual dimorphism in mean liability) already exists and consider whether a large‐effect mutation (or genomic inversion) can invade. This scenario could remove the initial requirement for appreciable heterozygosity at the large‐effect locus. Since sexual dimorphism in mean trait values can clearly evolve and show plasticity and there can also be sexual dimorphism in additive genetic and phenotypic variances (Lande 1980; Wyman and Rowe 2014), the background conditions for effective invasion of genetic variants that show SSDRs may be commonplace. Such scenarios for the evolution of SSDRs can be further examined in the future (see Discussion).

In the scenarios depicted in Figures 3, 4, 5, population‐wide distributions of liabilities are Gaussian, as is typically assumed in quantitative genetic analyses of threshold traits and is plausible given manifold genetic and environmental effects (Supporting Information S1; Wright 1934; Falconer and Mackay 1996; Lynch and Walsh 1998; Moorad and Promislow 2011). However, in principle, SSDRs in threshold traits could also readily emerge given different distributions of liabilities (even given uniform distributions, Supporting Information S3); the same principles as for Gaussian distributions still apply. Consequently, to generate SSDRs in threshold traits, there is no necessary condition that distributions of liabilities must be Gaussian or concave around the mean, as is required for genotype‐fitness maps to generate intrinsic SSDRs in sexually dimorphic traits that are directly expressed on observed phenotypic scales (Fry 2010; Connallon and Chenoweth 2019). Hence, overall, polygenic threshold traits involving large‐effect loci could readily generate substantial and dynamic SSDRs without requiring any specific or tightly restrictive underlying distributions of liabilities.

### GENOME‐WIDE SSDRs: SCENARIOS BASED ON EXPERIMENTAL EVOLUTION IN *DROSOPHILA*

While the above scenarios and salmonid examples concern SSDRs involving large‐effect loci, evidence of SSDRs that effectively involve highly polygenic variation, without any known loci of detectably large individual effects, has also emerged. Geeta Arun et al. (2021) undertook a major experiment that revealed polygenic SSDR for survival through exposure to bacteria (*Pseudomonas entomophila*) in *Drosophila melanogaster*. Such survival is clearly a key fitness component, implying selection for increased immunity. However, increased immunity may trade‐off against reduced mating success, particularly in males (Geeta Arun et al. 2021). Fitness may consequently be higher in less resistant individuals in the absence of bacterial exposure. Such trade‐offs may be weaker in females, which compete less strongly for mates. Immunity, and hence survival, can consequently experience sexually antagonistic selection of a magnitude that depends on bacterial exposure. Furthermore, in general, survival can often be reasonably envisaged as a highly polygenic threshold trait (Lynch and Walsh 1998; Moorad and Promislow 2011).

Geeta Arun et al.'s (2021) stock *Drosophila* showed low survival rates when experimentally challenged with *Pseudomonas*, with only slight (not statistically significant) phenotypic sexual dimorphism. Then, 65+ generations of experimental evolution, where parents in each generation comprised individuals who survived bacterial challenge, successfully generated lines that were more resistant, with much higher survival rates and still little phenotypic sexual dimorphism. This evolutionary response indicates substantial additive genetic variation underlying survival, with no evidence of sex linkage (Geeta Arun et al. 2021).

Geeta Arun et al. (2021) then crossed the evolved resistant lines back to the original stock and assayed the survival of the resulting ‘hybrid’ offspring through further experimental challenge with *Pseudomonas*. Sexual dimorphism then emerged, where female hybrids showed relatively high survival rates (closer to the resistant lines than the stock), while male hybrids showed relatively low survival rates (closer to the stock than the resistant lines). These patterns imply (partial) polygenic SSDR, at least assuming the hybrids are relatively heterozygous at numerous loci compared to the stock and resistant lines (Geeta Arun et al. 2021).

The question then is whether such SSDRs, with phenotypic sexual dimorphism in hybrid offspring without substantial phenotypic sexual dimorphism in either the stock or evolved parental lines, can potentially emerge in a threshold trait even without any explicit genetic dominance reversal (i.e., with purely additive genetic effects on liability). Simple scenarios suggest that they potentially can, due to the key property that mean phenotypic values of threshold traits depend on both the mean and variance in liability (Fig. 2).

**Figure 6:**
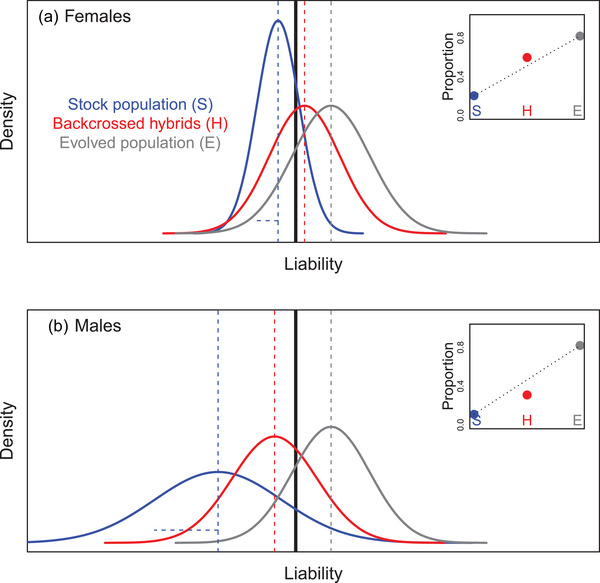
Illustration of emergence of (partial) sex‐specific dominance reversal (SSDR) in a threshold trait with sexual dimorphism in the mean and variance in baseline liability, following hypothetical experimental evolution and subsequent backcrossing. Blue curves show the distributions of baseline liabilities in the stock population in (A) females and (B) males. Gray curves show the distributions of liabilities following experimental evolution. Red curves show the distributions of liabilities in hybrids resulting from backcrosses between evolved and stock populations given purely additive genetic effects on means. Here, variance in liability might be slightly higher in males, depending on what mechanisms create and maintain sexual dimorphism in variance in the stock population. Black vertical lines denote the threshold, above which the focal trait (e.g., survival through bacterial exposure) is expressed. Accordingly, inset panels show the proportions of individuals of the stock (blue), evolved (gray) and hybrid (red) populations that express the phenotype (i.e., survive). There is little sexual dimorphism in either the stock or evolved populations, since similar proportions of the liability distributions exceed the threshold in both sexes. Dotted lines link the proportions for the stock and evolved populations, visualizing that the proportions for the hybrids lie above versus below the basic additive expectation in females versus males, representing partial SSDR. In the main figures, vertical dashed lines denote mean liabilities. Blue horizontal dashed lines highlight the standard deviations in liability in the stock population. There is therefore sexual dimorphism in both the mean and variance in liability in the stock population but not necessarily in the evolved population. Illustrated distributions are Gaussian, but this is not essential to generate SSDR. Parameter values are in Supporting Information S2

For example, consider that survival through bacterial challenge constitutes a threshold trait where the *Drosophila* stock population has some sexual dimorphism in both mean and variance in liability, such that males have lower mean and higher variance than females (e.g., blue curves on Fig. 6). This is broadly consistent with a stronger trade‐off between immunity and mating success in males than females, which could stabilize a lower mean yet maintain more (cryptic) genetic variation. However, despite such dual sexual dimorphism in baseline liability (i.e., in mean and variance), there may be little sexual dimorphism in the observed phenotypic survival rate (Fig. 6). Most liability values in both sexes lie below the threshold, implying relatively low survival rates (as observed by Geeta Arun et al. 2021).

Then, following experimental evolution, mean liabilities for both sexes lie above the threshold, with little or no sexual dimorphism in either mean or variance, or hence in mean phenotype (e.g., gray curves on Fig. 6). This outcome reflects that the experimental environment imposes consistent strong selection for increased immunity, potentially altering the balance of sex‐specific trade‐offs with mating success and decreasing the degree of sexual antagonism (as commonly postulated in harsher environments, Berger et al. 2014; Punzalan et al. 2014; Connallon and Hall 2016; Plesnar‐Bielak and Łukasiewicz 2021). Consequently, following evolution, most individuals of both sexes now survive (as observed by Geeta Arun et al. 2021).

Now, given crosses to create hybrid offspring and assuming purely additive genetic effects, mean liabilities for female and male hybrids could lie above and below the threshold, respectively, with some asymmetry (e.g., red curves on Fig. 6). The majority of females and males consequently survive and die, respectively. Mean phenotypic survival for the two sexes is therefore closer to the evolved versus stock parental lines, constituting (partial) SSDR (e.g., inset panels on Fig. 6, as observed by Geeta Arun et al. 2021).

This simple example illustrates how initial sexual dimorphism in the mean and variance in liability underlying a threshold trait can potentially generate apparent SSDRs following experimental evolution and backcrossing, even with purely additive genetic effects on the liability scale and without substantial phenotypic sexual dimorphism in either the stock or evolved populations. Such scenarios can be formally conceptualized in analogous ways as given a large‐effect locus, where the shift in mean breeding value generated by experimental evolution is analogous to the liability‐scale effect size of the alternative large‐effect allele (Supporting Information S1). The emergence of SSDRs therefore depends on the sex‐specific means and variances in liability in the baseline and evolved lines. Additional complexities could arise, for example, because variances in liabilities could more plausibly differ between lines than between groups of individuals who simply differ in genotype at a large‐effect locus (e.g., Fig. 6, Supporting Information S1).

However, scenarios such as that sketched in Figure 6 raise questions regarding the implications of such experimentally induced SSDRs for the maintenance of genetic variation and/or resolution of sexual conflict within focal populations in nature, which are key reasons why SSDRs are of interest. One immediate implication is that widespread expression of substantial net SSDRs for highly polygenic traits may require frequent introgression among diverged lines to generate relatively high degrees of genome‐wide heterozygosity in offspring of parents whose mean liabilities lie on opposite sides of the threshold. Such introgression could be relatively common in spatially structured systems where locally adapted populations are linked by dispersal. However, this is not the primary circumstance where additional explanations for the maintenance of genetic variation are needed. Rather, dispersal and resulting gene flow can directly maintain standing genetic variation exceeding that expected solely due to mutation‐selection balance (McDonald and Yeaman 2018). Furthermore, the persistence of local adaptation despite frequent introgression implies low fitness of hybrid offspring (or subsequent descendants). This in turn implies epistatic effects resulting in outbreeding depression or hybrid breakdown, which could eliminate phenotypic SSDRs. Consequently, it is not yet clear to what degree capacity for SSDRs as observed through experimental evolution and backcrossing among diverged lines will actually translate into substantial SSDRs in fitness in nature.

Increased genome‐wide heterozygosity within populations, and hence increased opportunity for SSDRs in highly polygenic traits, could also potentially be generated by disassortative mating for focal traits given positive cross‐sex genetic covariances in allelic effects. Such disassortative mating is conceivable in systems such as Geeta Arun et al. (2021) *Drosophila*, for example, if high‐immunity females are most likely to survive to reproduce while low‐immunity males are most attractive (given the postulated trade‐off, Geeta Arun et al. 2021). This would effectively represent positive assortative mating for fitness, given sexually antagonistic genetic effects (e.g., Arnqvist 2011). Some substantive degree of SSDR in fitness may then emerge in resulting offspring. However, more generally, some additional mechanism may be required to generate divergent sex‐specific phenotypic outcomes given the continuously distributed underlying genetic variation. Such mechanisms could potentially include the evolution of direct liability‐scale SSDRs or further nonlinearities resulting from competitive interactions.

### GENOME‐WIDE SSDRs: SCENARIOS BASED ON LINE CROSSES IN SEED BEETLES

The potential for competitive interactions to generate phenotypic SSDRs underpinned by polygenic variation can be considered with reference to the Grieshop and Arnqvist's (2018) line cross experiment in seed beetles. Here, strong sexually antagonistic selection occurs in the stock population, with a negative cross‐sex genetic correlation for fitness evident at standard temperatures (Berger et al. 2014). Grieshop and Arnqvist (2018) crossed 16 isogenic (inbred) lines representing the spectrum of female‐beneficial (male‐detrimental) versus male‐beneficial (female‐detrimental) variants in a full‐diallel design (i.e., all 16 lines mated with all 16 lines). Lifetime reproductive success (i.e., fitness) of the resulting F1 offspring was assayed through competitive trials. Then, for each of the 16 lines, the covariance between the mean competitive fitness of F1 offspring resulting from crosses between the focal line and each other line versus inbred F1 s from the other line was calculated (Grieshop and Arnqvist 2018). Here, small covariance implies that the focal line contains many dominant alleles, such that genetic effects of the other lines are effectively irrelevant. Conversely, large covariance implies that the focal line contains many recessive alleles, such that genetic effects of the other lines dominate. These covariances were calculated for females and males separately, and the cross‐sex correlation in covariances across the 16 lines was computed, giving a strongly negative value. Consequently, lines with small covariance across line crosses for males (implying genome‐wide dominance) had large covariance for females (implying genome‐wide recessivity), and vice versa. This implies genome‐wide SSDR for fitness (Grieshop and Arnqvist 2018, reviewed by Connallon and Chenoweth 2019).

Of interest here is the assay used to quantify individual fitness. To approximate natural conditions, Grieshop and Arnqvist (2018) staged competitive mating trials, where focal individuals competed against (sterilized) reference stock individuals for resources and fertilizations (females) or paternity (males). Such approximations of natural conditions are valuable since simple environments can strongly affect outcomes of sexual selection and associated experiments in model systems (Yun et al. 2017; Plesnar‐Bielak and Łukasiewicz 2021; Matzke et al. 2022). Indeed, the form of the fitness assay could potentially shape the manifestation of SSDRs by turning fitness into a threshold‐like trait.

**Figure 7:**
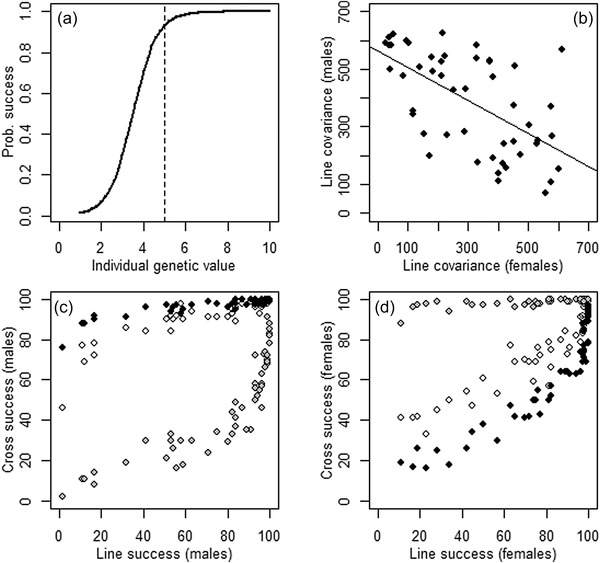
Summary of simulations that generate sex‐specific dominance reversals (SSDRs) in competitive fitness in a full diallel line‐cross given underlying additive genetic effects. (A) Form of the assumed nonlinear relationship between an individual's additive genetic value and its probability of paternity or maternity in competition with a reference individual. The depicted relationship is relatively extreme ‘winner takes all’, designed to illustrate key concepts. Simulations with less extreme relationships are shown in Supporting Information S4. The vertical dashed line indicates the mean genetic value of the simulated lines. (B) Emerging negative cross‐sex correlation between covariances between competitive fitness measured in F1 offspring of crosses between each focal line and each other line (i.e., cross success) versus F1 of the other line (i.e., line success). In the depicted simulation, the emerging correlation coefficient was strongly negative (−0.60). The solid line denotes the linear regression. (C, D) Illustrations of the relationships between cross success and line success for three representative focal lines (white, gray and black symbols) showing opposite covariances in (C) males versus (D) females. For example, the black‐symbol line shows a small line success versus cross success covariance in males but a large positive covariance in females, and these effects are reversed in the gray‐symbol line. These covariances form the points depicted in panel B across 50 simulated lines. Details of simulations and illustrative parameterizations are in Supporting Information S4

Specifically, if there is a negative cross‐sex genetic correlation in underlying additive genetic value and some degree of nonlinear or ‘winner takes all’ fitness outcome in both sexes, then females and males from female‐beneficial (i.e., male‐detrimental) lines will have disproportionately high and low success, respectively, while females and males from male‐beneficial (i.e., female‐detrimental) lines will have disproportionately low and high success, respectively. Simple simulations show how opposite nonadditive effects on fitness can then emerge in females versus males, readily generating a negative cross‐sex correlation in line cross covariance and hence apparent phenotypic SSDR (Figure 7, Supporting Information S4, as observed by Grieshop and Arnqvist 2018). Indeed, Grieshop and Arnqvist (2018) report some evidence of epistatic variance, which is consistent with a nonlinear fitness function.

Such outcomes depend on the shapes of the relationships between genetic value and competitive reproductive success in each sex and on the relative mean value of the reference population against which competitive reproductive success is assayed (which effectively defines the threshold for disproportionately high or low success, Fig. 7A, Supporting Information S4). The intrinsic emergence of SSDRs can consequently be shaped by details of the mating and reproductive systems, which therefore effectively act as dominance modifiers. Ongoing evolution of, or ecological or environmental effects on, the mating system and forms of pre‐ and postcopulatory sexual selection could consequently shape the manifestation of SSDRs in fitness.

However, the evolutionary implications of outcomes such as those observed in the seed beetle experiments (Grieshop and Arnqvist 2018) will again also depend on the degree to which heterozygosity across numerous loci affecting fitness actually arises in nature and the resulting degree to which the full intrinsic potential for SSDRs is actually expressed. Genome‐wide heterozygosity of magnitudes analogous to those resulting from inbred line crosses could plausibly arise in invertebrates and plants that can produce inbred or selfed generations on ephemeral or isolated resources followed by episodes of dispersal and outcrossing, generating cycles of inbreeding and outbreeding (e.g., Goodwillie et al. 2005; Cornell and Tregenza 2007; Whitehead et al. 2018), but will typically be more restricted otherwise. Intrinsic potential for genome‐wide SSDRs is therefore intertwined with mating system dynamics.

## Discussion

SSDRs could, in principle, contribute substantially to maintaining genetic variation and resolving sexual conflict in nature (Fry 2010; Barson et al. 2015; Spencer and Priest 2016; Grieshop and Arnqvist 2018; Connallon and Chenoweth 2019; Grieshop et al. 2021). However, the prevalence, magnitudes and implications of such effects depend on how SSDRs in fitness and fitness components actually arise in wild populations and hence on their potential for evolutionary, ecological and environmental modulation. I highlight how phenotypic SSDRs could in principle readily arise in threshold‐like traits characterized by dichotomous and/or competitive outcomes, potentially allowing rapid modulations that are intertwined with the evolutionary dynamics and plasticity of sexually antagonistic selection, sexual dimorphism and reproductive systems. New theoretical and empirical efforts are now required to examine the dynamics of locus‐specific and genome‐wide SSDRs arising in the contexts of natural genetic, ecological and environmental variation and to infer short‐term and longer‐term impacts on standing genetic variation and sexual conflict.

### EMERGENCE AND MODULATION OF SSDRS IN THRESHOLD(‐LIKE) TRAITS

The presented illustrative scenarios show how threshold(‐like) traits can readily generate phenotypic SSDRs that broadly caricature those observed in recent empirical studies, even given purely additive genetic effects on underlying scales. Given polygenic quantitative genetic architectures that include large‐effect loci, simply the presence of sexual dimorphism in mean baseline liability relative to the threshold (and potentially also in the variance) can generate phenotypic SSDRs (e.g., Figs. 3 and 4, Supporting Information S1). Given genome‐wide effects, substantial phenotypic SSDRs can emerge given crosses among diverged lines and/or given a negative cross‐sex genetic correlation and a reproductive system that generates some nonlinear or disproportionate outcome (e.g., Figs. 6 and 7, Supporting Information S4). Explicit SSDRs acting directly on underlying scales, for example, involving some form of direct sex‐specific genetic dominance modification, could exist but are not necessarily required and could conceivably even eliminate rather than generate phenotypic SSDRs (e.g., Fig. 5).

These scenarios show how forms of genetic and phenotypic sexual dimorphism and reproductive systems, which in turn shape and are shaped by the degrees of sexually antagonistic selection, can effectively act as broad‐sense dominance modifiers that could modulate the degree of phenotypic SSDR in threshold(‐like) traits. While the occurrence of sexual dimorphism in trait means is widespread and very well known, the possibility that there can be sexual dimorphism in genetic and/or environmental trait variances is also embedded in core aspects of evolutionary quantitative genetic theory and increasingly evidenced in diverse empirical systems, resulting from some degree of sex‐specific autosomal as well as sex‐linked genetic effects (e.g., Lande 1980; Brommer et al. 2007; Ober et al. 2008; Wyman and Rowe 2014; Janicke et al. 2016; Wolak et al. 2018; Kaufmann et al. 2021; van der Bijl and Mank 2021). Furthermore, degrees of sexual dimorphism, mating and reproductive systems, and magnitudes of sexually antagonistic selection and sexual conflict can commonly vary markedly with ecological and environmental conditions (e.g., Post et al. 1999; Punzalan et al. 2014; Taylor et al. 2014; Connallon 2015; Connallon and Hall 2016; de Lisle et al. 2018; Perry and Rowe 2018; Whitehead et al. 2018; Zhou et al. 2019; Chelini et al. 2021; Plesnar‐Bielak and Łukasiewicz 2021; Matzke et al. 2022). Indeed, numerous threshold traits, including alternative reproductive tactics, can show rapid environmentally induced expression of alternative phenotypes, implying environmental modulation (i.e., plasticity) on both liability and phenotypic scales (Roff 1996; Dodson et al. 2013; Neff and Svensson 2013; Reid and Acker 2022).

Taken together, these well‐established forms of sexual dimorphism and ecological variation imply that SSDRs in threshold(‐like) traits should not necessarily be viewed as fixed properties that could act as alternatives to evolved sexual dimorphism in resolving sexual conflict. Rather, they can be viewed as evolutionarily, ecologically, and environmentally labile outcomes that could emerge from, and potentially coevolve with, degrees of sexual dimorphism and reproductive systems. While it has long been established that dominance relationships emerge as intrinsic properties of nonlinear biological systems, such systems are often considered to be relatively fixed or stable (e.g., involving enzymatic and biochemical pathways and overall fitness landscapes generated by stabilizing selection, Otto and Bourguet 1999; Fry 2010; Manna et al. 2011; Connallon and Chenoweth 2019; Billiard et al. 2021, but see Gilchrist and Nijhout 2001). Considering the properties of threshold traits shows how biological systems that tune the degree of intrinsic SSDR could potentially be highly dynamic, readily evolve, and be subject to ecological and environmental modulation.

Such possibilities are pertinent because many key life‐history traits that affect fitness in wild, domesticated, and human populations can be reasonably conceptualized as polygenic threshold(‐like) traits. Obvious examples include the occurrence of maturation, seasonal migration, diapause, resistance to disease, survival, alternative reproductive tactics and the development of alternative morphologies (Roff 1996; Moorad and Promislow 2011; Pulido 2011; Dodson et al. 2013; Neff and Svensson 2013; Wray and Visscher 2015; Debes et al. 2021; Reid and Acker 2022). Forms of mate choice, competition and resulting sexual selection can also readily generate nonlinear relationships between phenotypic trait values (and hence underlying additive genetic effects) and fitness. Such nonlinearities arise where single individuals dominate mating or reproduction (i.e., strongly skewed outcomes of intrasexual competition), where all or most individuals preferentially mate with the same chosen mate(s) (i.e., strongly directional pre‐ or postcopulatory mate choice); and/or single males achieve disproportionate fertilization success through postcopulatory processes (e.g., ‘loaded raffle’ outcomes of sperm competition and/or strong first‐ or last‐mating precedence). Some degree of ‘winner takes all’ is consequently commonplace across diverse taxa and reproductive systems (e.g., Nonacs and Hager 2011; Dodson et al. 2013; Laturney et al. 2018; Parker 2020; Matzke et al. 2022), including in the seed beetles that generated evidence of genome‐wide SSDRs (Yamane et al. 2015). Fitness will therefore typically be affected by at least one threshold(‐like) trait in many, or most, species.

Accordingly, the potential for threshold‐like traits to generate strong phenotypic SSDRs, including through coevolutionary feedbacks involving genetic architectures, forms of sexual dimorphism and reproductive systems, should now be more explicitly examined, both theoretically and empirically. Such work can aim to more clearly distinguish key points: the degrees to which SSDRs can in principle arise through combinations of intrinsically nonlinear genotype–phenotype maps and/or explicit genetic dominance modification and the degrees to which such SSDRs are actually likely to be expressed, to be dynamic and to act as predominant forces that could widely maintain genetic variation and resolve sexual conflict given forms and impacts of heterozygosity arising in nature.

### OPPORTUNITIES FOR THEORETICAL ADVANCES

Multiple opportunities for theoretical advances are evident. First, we can examine whether, by facilitating emergence of phenotypic SSDRs, threshold traits with sexual dimorphisms in liability could actually facilitate invasion and maintenance of stable polymorphisms for large‐effect mutations or complexes of linked genes with sexually antagonistic phenotypic effects. We can then examine whether such invasions can feed back to shape the form and plasticity of underlying sexual dimorphism. To date, the dynamics of genetic architectures involving large‐effect loci or gene clusters have been examined in the context of local adaptation and migration‐selection‐drift balance (e.g., Yeaman and Whitlock 2011; Yeaman 2013) but scarcely explicitly considered in the context of threshold traits or SSDRs (or more widely in the context of balancing selection, Llaurens et al. 2017). Such work would encompass the key point that, since phenotypic dominance of any large‐effect allele effectively depends on (polygenic) genetic values for baseline liabilities, SSDRs in threshold traits can substantially reflect epistasis. While it has been highlighted that forms of additive‐by‐additive epistasis can shape the maintenance of sexually antagonistic genetic variation given SSDRs (Arnqvist et al. 2014), the reciprocal point that intrinsic SSDRs in threshold(‐like) traits can effectively result from epistasis given underlying sexual dimorphism has not been emphasized.

Second, we can examine whether, given initial sexual conflict manifested as negative cross‐sex genetic correlations for fitness, reproductive systems can actually evolve to shape fitness functions that generate some degree of disproportionate competitive outcome and thereby generate SSDRs that in turn ameliorate sexual conflict. Such evolution could, for example, conceivably involve diverse mechanisms that shape the occurrence and outcome of competition for reproduction, including degrees of directional mate choice, first‐ or last‐mating precedence, and even polyandry itself. Expression of substantial genome‐wide SSDRs shaped by numerous loci of small effect also requires some degree of genome‐wide heterozygosity, which could be fostered by evolution of some degree of disassortative mating for traits (and resulting assortative mating for fitness given sexual antagonism, Arnqvist 2011). However, by imposing sexual selection for opposite sex‐specific phenotypes, the evolution of disassortative mating could potentially exacerbate net sexually antagonistic selection on target phenotypes and result in sexual conflict. The evolution of mechanisms that generate heterozygosity and thereby foster SSDRs could therefore conceivably strengthen rather than necessarily reduce conflict, potentially undermining any evolutionary benefit of SSDRs. Any such joint dynamics of SSDRs and reproductive systems could also usefully be placed in the context of population structure and environmental variation and change, which can alter the degrees of heterozygosity and sexual conflict and shape sex‐specific evolutionary outcomes (e.g., Berger et al. 2014; Punzalan et al. 2014; Connallon and Hall 2016; de Lisle et al. 2018; Perry and Rowe 2018; Chelini et al. 2021; Plesnar‐Bielak and Łukasiewicz 2021; Tschol et al. 2022).

Third, we can examine the plausibility of the evolution of explicit dominance modifiers that act directly on underlying liability scales. In general, loci affecting liabilities could potentially show relatively high heterozygosity, which is generally required for the evolution of dominance modifiers (e.g., Otto and Bourguet 1999; Spencer and Priest 2016). This is because such loci can maintain a relatively high mutation‐selection‐drift balance (Roff 1998), which in turn is because genetic variants typically have no phenotypic effect if occurring in a liability background that is far from the threshold and are consequently sheltered from selection. However, by the same logic, any liability‐scale SSDRs will not necessarily be phenotypically expressed (e.g., Fig. 5), meaning that otherwise neutral dominance modifiers will not be subject to (indirect) selection. It is consequently unclear to what degree, or under what circumstances, direct liability‐scale dominance modifiers in threshold(‐like) traits could evolve.

Overall, therefore, there is considerable scope for evolutionary dynamics of both phenotypic and liability‐scale SSDRs in threshold(‐like) traits to be formally considered through new models that jointly track the (co)evolution of multiple routes to generate and resolve sexual conflict, including sexual dimorphisms and complex reproductive systems.

### OPPORTUNITIES FOR EMPIRICAL ADVANCES

There is also considerable scope for future empirical studies to explicitly examine the basis and modulation of phenotypic SSDRs in threshold(‐like) traits. First and most obviously, we should more explicitly distinguish whether observed phenotypic SSDRs (or lack of SSDRs) result from direct SSDRs acting on underlying liability scales, from the properties of defined nonlinear genotype‐phenotype or genotype‐fitness maps (given purely additive underlying genetic effects), or both. This distinction requires estimating appropriate liability‐scale fixed effects and variance components and back‐transforming onto observed phenotypic scales, which has not yet been a primary focus of empirical studies of SSDRs in threshold traits (Supporting Information S5, but see Debes et al. 2021). Such analyses can be enacted using established machineries of generalized linear mixed models (GLMMs), which intrinsically distinguish liability and observed scales and where algorithms for back‐transforming fixed effects and random effect variances (and variances conditional on fixed effects) are available (de Villemereuil et al. 2016). Specifically, GLMMs with binomial error distributions and probit link functions correspond to the threshold trait model.

Second, we should more explicitly quantify the degree to which phenotypic SSDRs are modulated by ecological and environmental conditions, thereby treating SSDRs as dynamic rather than fixed entities. This could be achieved, for example, by manipulating environmental conditions that affect the degree of sexually antagonistic selection or the degree or form of competition for reproductive success. Any experimental design to reveal SSDRs requires major efforts, even without any ambition to replicate across different conditions (e.g., Grieshop and Arnqvist 2018). However, experiments designed to quantify variation in SSDRs could potentially be streamlined, for example, by focusing on fewer targeted crosses experiencing different environments.

Third, we should more extensively quantify the frequency, dynamics, and net magnitude of SSDRs arising in threshold(‐like) traits in wild populations. This objective will require attention to how locus‐specific or genome‐wide heterozygosity arises and to the overall phenotypic consequences of such heterozygosity. It has been widely emphasized that substantial heterozygosity is required for the evolution of dominance modifiers (Otto and Bourguet 1999; Spencer and Priest 2016; Connallon and Chenoweth 2019). However, even when SSDRs arise as intrinsic properties of threshold(‐like) traits (or other nonlinear systems with purely additive underlying genetic effects, e.g., Gilchrist and Nijhout 2001; Fry 2010; Vasseur et al. 2019) where initial heterozygosity is not required for system evolution, SSDRs will not be maximally expressed and hence will have reduced biological impact if there is little heterozygosity across contributing loci. The two recent studies that demonstrated SSDRs in free‐living salmonids focused on large‐effect sexually antagonistic loci (Barson et al. 2015; Pearse et al. 2019), greatly facilitating direct identification and comparison of heterozygotes and homozygotes. However, such architectures may generally be more exceptional than typical. SSDRs involving highly polygenic variation, where individual loci have very small liability effects that may not be directly phenotypically expressed, should now be explicitly examined in wild or wild‐derived populations in at least seminatural environmental conditions. This could potentially be achieved by quantifying sex‐specific fitness of known offspring of immigrant‐native crosses relative to parental populations in structured meta‐population systems or of individuals with different degrees of genome‐wide heterozygosity resulting from local inbreeding versus outbreeding. The emergence of SSDRs can then be evaluated in the context of architectures that shape the relative fitness of polygenic heterozygotes and homozygotes, notably the degree of directional dominance (which underpins both inbreeding depression and heterosis, Falconer and Mackay 1996; Lynch and Walsh 1998). Such data on genome‐wide heterozygosity and fitness components will be increasingly available through multigeneration individual‐based and/or genomic studies of wild populations and should be central to ascertaining the potential and actual impacts of dynamic SSDRs in maintaining genetic variation and resolving sexual conflict in nature.

## AUTHOR CONTRIBUTIONS

J.M.R. conceived the ideas and wrote the manuscript.

## DATA ARCHIVING

There are no primary data associated with this conceptual manuscript. The R code underlying the illustrative figures is provided as Supporting Information.

Associate Editor: T. Connallon

Handling Editor: T. Chapman

## Supporting information

S1. Derivations of SSDRs given a threshold trait with a large‐effect locus
S2. Parameter values
S3. Illustration of the emergence of SSDRs given uniform distributions of liabilities
S4. Emergence of genome‐wide SSDRs in a full diallel line‐cross
S5. Empirical estimation of SSDRs

Supplementary information
